# Learning to Predict Page View on College Official Accounts With Quality-Aware Features

**DOI:** 10.3389/fnins.2021.766396

**Published:** 2021-10-28

**Authors:** Yibing Yu, Shuang Shi, Yifei Wang, Xinkang Lian, Jing Liu, Fei Lei

**Affiliations:** ^1^The Communist Youth League Committee, Beijing University of Technology, Beijing, China; ^2^School of Economics and Management, Beijing University of Technology, Beijing, China; ^3^Faculty of Information Technology, Beijing University of Technology, Beijing, China

**Keywords:** page view, quality-aware features, selective ensemble, human visual system, college official accounts

## Abstract

At present, most of departments in colleges have their own official accounts, which have become the primary channel for announcements and news. In the official accounts, the popularity of articles is influenced by many different factors, such as the content of articles, the aesthetics of the layout, and so on. This paper mainly studies how to learn a computational model for predicting page view on college official accounts with quality-aware features extracted from pictures. First, we built a new picture database by collecting 1,000 pictures from the official accounts of nine well-known universities in the city of Beijing. Then, we proposed a new model for predicting page view by using a selective ensemble technology to fuse three sets of quality-aware features that could represent how a picture looks. Experimental results show that the proposed model has achieved competitive performance against state-of-the-art relevant models on the task for inferring page view from pictures on college official accounts.

## 1. Introduction

With the popularization and development of the Internet, the official accounts have attracted extensive attention. The majority of college departments now own accounts because it has become the main channel for publishing notices and posting news. Page view is a very significant indicator for college official accounts, capable of visually showing the popularity of an article. If we can predict the page views, it is of great help to improve the attention of audience for articles. The number of views on articles is influenced by the content of pictures. To this end, we explore the quality-aware features of pictures and attempt to predict page views in the official accounts based on image processing technology in this paper.

In recent years, with the development of image processing technology, there are many great contributions in multimedia telecommunication domain (Geng et al., 2011; Kang et al., 2019; Moroz et al., 2019; Su et al., 2019; Wu et al., 2019; Yildirim, 2019), education and teaching (Richard, 1991; Greenberg et al., 1994; Rajashekar et al., 2002; Yaman and Karakose, 2016), and environmental perception and protection, such as air pollution detection (Gu et al., 2020a,c, 2021b; Liu et al., 2021), PM_2.5_ monitoring (Gu et al., 2019, 2021a), air quality forecast (Gu et al., 2018, 2020b), and distance education (Zheng et al., 2009). Among them, picture quality assessment (PQA) has been receiving a lot of attention as an important part of image processing technology. With a variety of PQA models available from Wang et al. (2004), how to achieve evaluation results that are consistent with the subjective PQA of human beings is crucial. Usually, subjective experiments are performed by human observers who score the pictures, and the final reliable results obtained are taken as the ground truth (Gu et al., 2014, 2015a). However, the method mentioned above is time consuming and complicated, so the focus of relevant scientific research has shifted to the design of objective PQA algorithms implemented by computers. The objective PQA algorithm has the characteristics of convenience, high-speed, repeatable, batch processing, and real-time, which make up for the deficiency of the subjective PQA method.

The objective PQA approach establishes a mathematical model that is combined with the subjective human visual system (HVS) to realize the evaluation of picture quality. According to the amount of information provided by the reference picture, the existing objective PQA methods can be divided into: full reference (FR) PQA method, reduced reference (RR) PQA method, and no reference (NR) PQA method. Among them, the FR PQA method is the most reliable and technically mature evaluation method. It has a complete original picture and allows a one-to-one correspondence comparison of the distorted picture with the pixels of the original picture. Instead, RR PQA method requires only partial original picture information, researchers like Liang and Weller (2016) and Wu et al. (2013) put forward a series of novel RR PQA algorithms. The FR PQA algorithm and the RR PQA algorithm combine the visual features of the picture to quantify the difference between the original picture and the distorted picture, so as to get the quality of pictures.

In official accounts, the original picture information is not available, so it is particularly important to propose PQA algorithm. Most of the current NR PQA methods were proposed based on two steps, which are feature extraction proposed by Gu et al. (2017b) and the support vector machine (SVM) proposed by Smola and Schölkopf (2004) that can find out the underlying relationship between the selected features and human subjective evaluations. No reference method is a situation where none of the information contained in any reference picture or video is used to draw quality conclusions. Since the picture is not available in most cases, more and more metrics were proposed for NR PQA method. Nowadays, the advanced method (e.g., BRISQUE) is a universal blind PQA model based on Natural Scene Statistics (NSS) proposed by Mittal et al. (2012). Natural scene pictures belong to a small domain of Internet picture signals that follow predictable statistical laws. Specifically, the natural scene pictures captured by high-quality devices obey the Gaussian-like distribution, while the pictures with distortion (such as blur, noise, watermarks, color transformation, etc.) do not follow the Bell curve law. Based on this theory, the features of NSS can be used as an effective and robust natural PQA tool. In recent years, a large number of studies based on NSS have been carried out, such as the MSDDs presented by Jiang et al. (2018), Bliinds-II constructed by Saad et al. (2012), BLIQUE-TMI created by Jiang et al. (2019b), GMLF designed by Xue et al. (2014), and DIIVINE presented by Moorthy and Bovik (2011), which is capable of assessing the quality of distorted pictures across multiple distortion categories, etc. In addition, Ruderman (1994) investigated the data rules of natural pictures, which provides a basis for evaluating the perceptual quality of pictures. The local features of pictures can perfectly reflect the perceptual quality of pictures.

Due to the fact that most of the audiences get the information from official accounts from vision, we also introduce into the approach based on the HVS. Advances in brain science and neuroscience studied by Friston et al. (2006) have encouraged scholars to explore new fields of machine vision. Eye movement research is also of significance to the visual perception of brain science. Jiang et al. (2019a), Kim et al. (2019), Lin et al. (2019), Tang et al. (2020), Zhang et al. (2020), Jiang et al. (2021), Wang et al. (2021) had carried out a lot of research work. Brain science research have shown that the brain produces an intrinsic model to explain the process of perception and understanding, and that the free energy generated during this cognitive process can reflect the difference between picture signals and internal descriptions. By modeling important physiological and psychological visual features, Xu et al. (2016) discussed the mechanism related to free energy in the human brain and proposed an efficient PQA method by using JPEG and JPEG2000 compression, Jiang et al. (2020) presented a new FR-SIQM method by measuring and fusing the degradations on hierarchical features. Besides, Gu et al. (2015b) designed the NFSDM in an alternative way of extracting features. On the basis of the NFSDM approach, the NFERM is combined with HVS to reduce the number of extracted by half, further improving the accuracy of the evaluation.

Based on image processing technology, this paper investigates a large collection of quality-aware features of pictures to predict the page view that reflects the popularity of articles. To accomplish this goal, the authors do a lot of work to collect the pictures published by the WeChat official accounts of nine universities in Beijing in recent months, and establish a new picture database consisting of 1,000 pictures. In addition, we collect three groups of features from the Official Accounts Picture Quality Database (OAPQD) and use the selective ensemble technique proposed for NSS, HVS, and histogram feature analysis to fuse these features, allowing them to fit the correlation between page view and the quality of pictures. The results of experiments show that these features are able to predict the page view of articles, and that the method of using the three groups of features can more accurately fit the correlation.

The structure of this paper is as follows. In section 2, we describe the construction of the OAPQD dataset. In section 3, the three features and the selective ensemble method that can fuse them are presented separately. We conduct the comparison experiment on the OAPQD to analyze the magnitude of the seven features on fitting the page view in section 4. Section 5 gives the concluding remarks.

## 2. The Dataset

**Figure 1:**
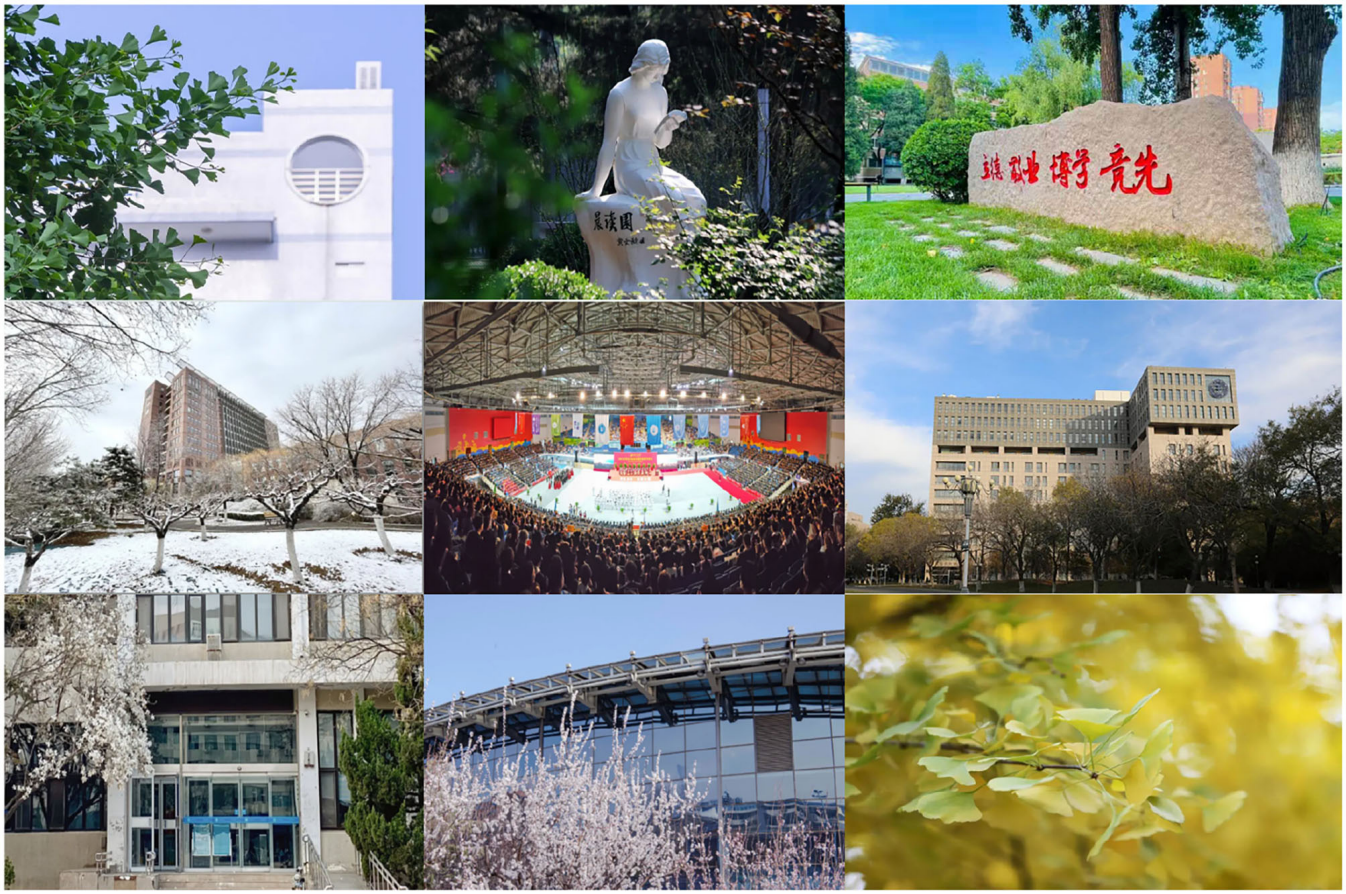
Representative nine pictures from the OAPQD data set, the content of above mainly includes architecture, landscape, people, text content, meeting scene, etc.

With the development of information and network technology, traditional media were gradually replaced by digital new media, such as WeChat official account, which has been widely used by all walks of life. Currently, most universities use official accounts as the platform for campus culture construction. In order to better explore the reasons why articles are popular on public accounts, we focus mainly on the page view of articles. To this end, we first subscribed to the WeChat official accounts of nine well-known universities in Beijing, then selected the pictures inserted in the articles that were published by the accounts in the past months, based on which a new database is created. To be specific, the most researched and representative pictures are extracted from the selected article. Simultaneously, the number of page views corresponding to the selected article is recorded, with a maximum of 100,000 and a minimum of 253. We selected a picture from a large number of articles published by official accounts of schools every day, and we have collected 1,276 pictures altogether. However, not each of the above pictures has research value. In these pictures, the selection criteria are first based on the picture content and type, and then exclude extreme special cases, such as the case where the picture quality is very poor but the number of clicks is very high. Finally, 1,000 most representative pictures were selected to form the picture data set. Figure 1 shows the subset of OAPQD.

**Figure 2:**
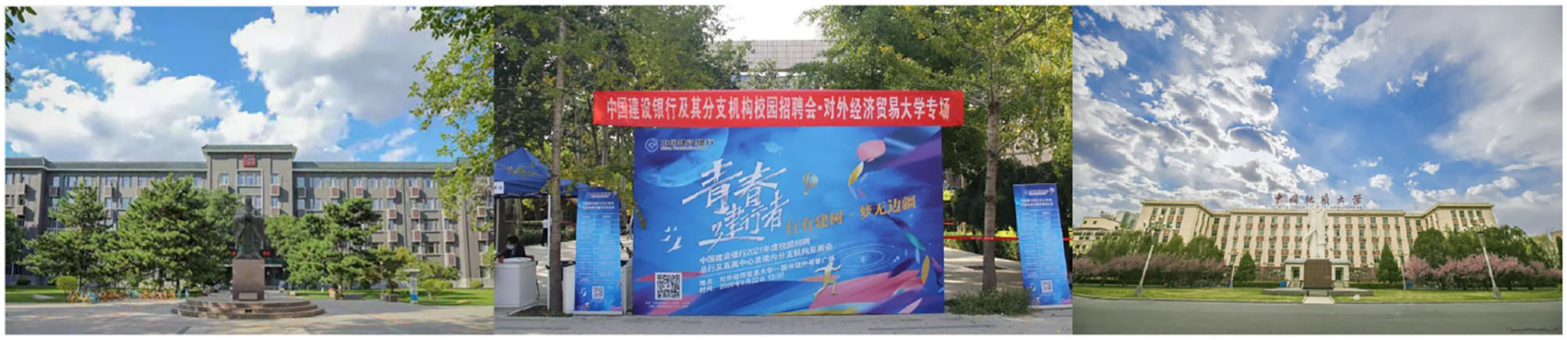
The quality of the three pictures in the OAPQD decreases gradually from left to right.

By observing the data set we constructed, we find that there is a positive correlation between picture quality and page view. As shown in Figure 2, there are three pictures from left to right. The picture on the left is the most colorful and clear among the three pictures, giving a better visual experience with 41,000 hits. The intermediate picture is of poor quality, with only 7,466 clicks. The picture on the far right is the least visually appealing and thus logically the least clicked picture with only 1,052.

## 3. Methodology

The specific features can well reflect the page view of pictures, but the fitting accuracy of using a certain characteristic feature alone is relatively low. In this section, we will introduce the three groups of complementary features extracted based on natural scene analysis, histogram, and free energy theory, and further describe a selective ensemble approach capable of fusing the 99 features.

### 3.1. NSS-Based Feature Extraction

The first group is composed of 36 features (f_01_-f_36_), which were proposed on the basis of NSS theory. Bovik (2010) suggested that natural pictures have regular statistical characteristics, therefore, the statistical features of natural scenes can be considered as an effective and powerful tool for PQA. In general, complex image textures affect the perceptual level of distortion, and the local brightness normalization can greatly reduce the correlation between adjacent pixels of the original picture and the distorted picture. Thus, the classic spatial NSS model is first used to preprocess the picture to remove the local mean value, and then the picture is segmented and normalized to extract the mean subtracted contrast normalized coefficient of natural scene pictures. The Mean Subtracted Contrast Normalized (MSCN) coefficients vary in different ways due to distinct distortions. On the basis of this variation, the type of picture distortion and the perceived quality of pictures can be predicted. The pixel intensity of natural pictures follows a Gaussian distribution, which can be represented by a Bell curve. In order to clearly observe the differences in data distribution between different distortion types and natural pictures, we use the generalized Gaussian distribution (GGD) to fit the distribution of MSCN. The sign of the transformed picture coefficients are regular, but Mittal et al. suggested that the existence of distortion affects this above correlation structure. In order to research the correlation information between connected pixels, the zero-mode AGGD is used to model the inner product of MSCN adjacent coefficient. The moment matching-based approach proposed by Lasmar et al. (2009) can estimate the parameters of the AGGD. Then we calculate the adjacent pairs of coefficients from the horizontal, vertical, and diagonal directions to obtain the 16 parameters, respectively. Low-resolution pictures are obtained from each picture through low-pass filtering and downsampling with a factor of 2. We measure the MSCN parameters fitted by GGD and the 16 parameters generated by AGGD according to the above two scales. Once all the work mentioned above is done, the first feature set consisting of 36 features is obtained.

### 3.2. Histogram-Based Feature Extraction

The second group consists of 40 features (f_37_-f_76_), illustrating the main features of the HVS introduced from biology in image processing. Since the visual information in picture is often redundant, the understanding of the HVS is mainly related to its basic features, such as contour, zero cross, and so on. Gradient magnitude (GM) feature can reflect the intensity of local luminance variations. The local maximum GM pixels can reflect small details and textural change of pictures, which is the main element of contour. GM has been widely used for PQA methods, such as FSIM proposed by Zhang et al. (2011), GMSD constructed by Xue et al. (2013), PSIM designed by Gu et al. (2017a), and ADD-GSIM established by Gu et al. (2016), where picture quality is evaluated only by the similarity of gradient magnitude. Besides, on the basis of GM method, Min et al. (2019b) first proposed a picture dehazing algorithm, then a novel objective index named DHQI was presented by Min et al. (2019a) can be utilized to evaluate DHAs or optimize practical dehazing systems. Finally, a blind PQA method was introduced by Min et al. (2018) has a superior performance. Generally, GM is calculated using linear filter convolution, where the typical filters are mainly Sobel, Prewitt, Roberts, etc. Unlike the GM operator, isotropic measurements on the second spatial derivative of pictures show the strongest brightness variation. The Laplacian of Gaussian (LOG) operator reflects the intensity contrast of a small spatial neighborhood, and Marr and Hildreth (1980) proposed that it can model the receptive fields of retinal ganglion cells. The LOG operator and the GM operator adopt the anisotropic calculation method without angular preference to obtain the local picture structure from different angles. They can represent the structural information of pictures, especially the local contrast features, and therefore can be used to form the semantic information of pictures. Finally, the picture local quality prediction is achieved by using these two operators mentioned above.

### 3.3. Free Energy-Based Feature Extraction

The 23 features (f_77_-f_99_) extracted in the third group are inspired by the free energy principle and the structural degradation model (SDM). A basic premise of the free energy theory is that an internal generative model can be used to estimate the gap between the viewing scene and the corresponding brain prediction. It measures the difference between the probability distribution of environmental quantities acting on the system and an arbitrary distribution encoded by its configuration. Since this process is very closely related to the quality of human visual perception, it can be used for the PQA method. The free energy of pictures can be approximated by the AR model as the total description length of pictures data. In an effective RR SDM proposed by Gu et al. (2015b), we observe the structural degradation after low-pass filtering of the picture. The spatial frequency of input picture *I* has different degrees of decrease. We first define the local mean and variance of *I* with a two-dimensional circularly symmetric Gaussian weighting function. The linear dependence between the free energy and the structural degradation information provides an opportunity to characterize distorted pictures in the absence of the information of original picture. Furthermore, the NFEQM is added to the third group as feature f_99_ due to its excellent performance in noisy and blurred pictures.

### 3.4. Selective Ensemble-Based Page View Inference

A single picture feature does not represent the picture quality well, which will lead to its poor fitting of the relationship between features and page view. To solve this question, we consider an ensemble learning approach which can produce strong generalization to improve the fitting accuracy. This content has become a hot research topic in the international machine learning community, so there are more and more methods presents by scholars, such as the geometric structural ensemble (GSE) learning framework approach presented by Zhu et al. (2018). Zhou et al. (2002) suggested that the presence of high-dimensional selective ensemble methods based on direct merging is prone to overfitting or some of these features may be overlooked in the fitting process. On the basis of this theory, we adopt the method of selective ensemble to further enhance the performance of our presented approach in this paper.

It is natural to combine features to derive a more effective preprocessing method, so as to better remove random details caused by the varying viewing method and picture resolution in different but supplementary domains. We combine the three features two by two and last fuse the three by using a selective ensemble technique proposed by Gu et al. (2020b) and Chen et al. (2021), so as to make an experimental comparison with the accuracy of the fit using single features. The following seven categories can be generated based on the number of features: (1) BRISQUE; (2) GMLF; (3) NFERM; (4) BRISQUE+GMLF; (5) BRISQUE+NFERM; (6) GMLF+NFERM; (7) BRISQUE+GMLF+NFERM. The experimental results show that the number of fused features affects the linearity of the results, where the method that fuses three features has the best performance and the single feature has the worst accuracy in fitting the correlation between picture quality and page view.

## 4. Experimental Results and Analysis

In this section, we carry out the comparison experiment on the OAPQD, so as to understand the degree of seven features on fitting the page view of articles. In this process, we select the two classical metrics to evaluate the performance of experiments.

In order to further predict page view with quality-aware features, experiments are conducted on the OAPQD dataset consisting of 1,000 pictures selected from the WeChat official accounts of nine universities in Beijing. The pictures from the dataset used for testing are rich in content and variety, and can improve well the hit of pictures published in the college official accounts. This provides a certain foundation for our proposed method. In the experimental analysis section, we use two commonly statistical indicators as the metrics to assess the performance, which are the Pearson linear correlation coefficient (PLCC) and the Spearman rank order correlation coefficient (SRCC). The PLCC is a linear correlation coefficient with scale invariance, which indicates the degree of similarity between picture features and page view. The PLCC is defined as


(1) $$PLCC=\frac{\sum_{i}\left(q_{i}-\bar{q}\right)\cdot (o_{i}-\bar{o})}{\sqrt{\sum_{i}{\left(q_{i}-\bar{q}\right)}^{2}\cdot \sum_{i}{\left(o_{i}-\bar{o}\right)}^{2}}}$$

where *o*_*i*_ and $\bar{o}$ represent the features of the *i*th picture and its overall mean value, and *q*_*i*_ and $\bar{q}$ are the page view of *i*th picture and its mean value. Before using the PLCC metric for evaluation, we employ the nonlinear regression equation proposed by Sheikh et al. (2006), which is given by


(2) $$\begin{array}{r} p\left(x\right)=\alpha_{1}\left[\frac{1}{2}-\frac{1}{1+e^{\alpha_{2}\left(x-\alpha_{3}\right)}}\right]+\alpha_{4}x+\alpha_{5} \end{array}$$

where *p*(*x*) represents the predicted score, α_*i*_ (*i* = 1,2,3,4,5) is the parameter of the generation fitting, and *x* is the original prediction score. While the SRCC represents the strength of the monotonic relationship predicted by the algorithm, it can be calculated by


(3) $$\begin{array}{r} SRCC=1-\frac{6}{N\left(N^{2}-1\right)}\overset{N}{\underset{i=1}{\sum }}d_{i}^{2} \end{array}$$

where *N* is the number of pictures in the dataset, and *d*_*i*_ is the difference between the ranking of *ith* picture in features and page view. The value range of PLCC and SRCC is [−1, 1]. The closer the absolute value of these two indicators is to 1, the stronger the correlation between picture features and page view, where >0 means a positive correlation and <0 means a negative correlation. In the regression problem, the closer the value is to 1, the higher the accuracy of the algorithm.

We extract three types of features, where the first set of feature coefficients has the characteristic statistical property of varying due to the distortion. Quantifying these variations allows obtaining the type of picture distortion while enabling the prediction of page view. The second group of features is composed of 40 local contrast features, GM and LOG, which can detect changes in the semantic structure of the picture due to variations of luminance for the purpose of predicting the page view of the article. The third set of features consists of 23 features based on free energy and structure degradation information. In addition, they are inspired by the HVS and the free energy theory, which fill the gap in the NR PQA method due to the lack of prior knowledge.

The features mentioned above can reflect the page view well, and based on this, we use selective ensemble technology to fuse features in different ways for comparison experiments. The results of comparison experiments show that the method that fuses all the three features together obtain the largest data value and the highest accuracy of the results, followed by the method of fusing two features. The experimental data is placed inside Table 1, where the values obtained by the best-performing method are given in bold. In Table 1, it can be seen that the values of PLCC and SRCC are very approximate when using a single algorithm. It is not difficult to find that GMLF has gained the best results (on average) of SRCC, which is sensitive to pictures with gradient features. However, Table 1 reports the low correlation performance on SRCC when combined with the features from BRISQUE and NFERM. It also can be seen that the more the number of fused picture features, the better the fit to the relation between features and page view. Meanwhile, it shows a certain degree of similarity between the features and click-through rate. This method proposed in this paper can provide guidance for the management of college official accounts. For example, the insertion of high-definition and high-quality pictures into published articles can increase the visibility of the articles.

**Table 1:** The Pearson linear correlation coefficient (PLCC) and Spearman rank order correlation coefficient (SRCC) values of seven feature fusion methods on the dataset.

**Algorithm**	**PLCC**	**SRCC**
BRISQUE (direct use)	0.0156	0.0347
GMLF (direct use)	0.0034	0.0343
NFERM (direct use)	0.0683	0.0146
BRISQUE (re-train)	0.3925	0.2707
GMLF (re-train)	0.3911	0.3340
NFERM (re-train)	0.3983	0.2782
BRISQUE+GMLF	0.4545	0.3577
BRISQUE+NFERM	0.4454	0.3054
GMLF+NFERM	0.4387	0.3655
BRISQUE+GMLF+ NFERM	**0.4764**	**0.3863**

*The top data values are given in bold*.

## 5. Conclusion

In this paper, we have studied the connection between picture features and the popularity of articles published in college official accounts. We elaborately select 1,000 pictures from the official accounts of nine universities, construct a picture database named OAPQD, and record the clicks of corresponding articles. Three groups of features extracted from different angles can reflect the features, and the stacked selective ensemble technology is used to fuse them for comparison experiments. The experimental results show that the method integrating three groups of 99 features at the same time has the highest accuracy in fitting the page view. Therefore, in future publicity work, the selection of pictures is very meaningful for the popularity of official account articles. For the publicity department of the college, they can import our method to predict the page views of their articles and use these data parameters to adjust picture quality or change diffusion strategy. All of these measures can improve the visibility of official accounts to some extent.

## Data Availability Statement

The raw data supporting the conclusions of this article will be made available by the authors, without undue reservation.

## Author Contributions

YY conceived the framework of the paper and completed the main content of the paper. SS collected a large number of references to provide a strong background basis for the paper. YW was mainly responsible for the revision of the thesis. XL participated in the revision and content supplement of the article. JL revised the layout of the article and checked for grammatical errors. FL checked the final version of the paper. All authors contributed to the article and approved the submitted version.

## Funding

This work was supported by National Social Science Foundation of China (15BJY048).

## Conflict of Interest

The authors declare that the research was conducted in the absence of any commercial or financial relationships that could be construed as a potential conflict of interest.

## Publisher's Note

All claims expressed in this article are solely those of the authors and do not necessarily represent those of their affiliated organizations, or those of the publisher, the editors and the reviewers. Any product that may be evaluated in this article, or claim that may be made by its manufacturer, is not guaranteed or endorsed by the publisher.
